# A tale of two caspases

**DOI:** 10.7554/eLife.106581

**Published:** 2025-03-31

**Authors:** Denise M Monack

**Affiliations:** 1 Department of Microbiology and Immunology, Stanford University School of Medicine Stanford United States

**Keywords:** *Salmonella enterica* serovar Typhimurium, inflammasomes, human macrophages, Human, Other

## Abstract

Macrophages control intracellular pathogens like *Salmonella* by using two caspase enzymes at different times during infection.

**Related research article** Egan MS, O’Rourke EA, Mageswaran SK, Zuo B, Martynyuk I, Demissie T, Hunter EN, Bass AR, Chang YW, Brodsky IE, Shin S. 2023. Inflammasomes primarily restrict cytosolic *Salmonella* replication within human macrophages. *eLife* **12**:RP90107. doi: 10.7554/eLife.90107.

*Salmonella* bacteria are well-known (and feared) for causing countless foodborne illnesses. After entering the body through contaminated food, *Salmonella* target the intestinal tract, where they invade and replicate in host cells, including frontline defenders called macrophages (Santos and Bäumler, 2004).

The bacteria use a syringe and needle-like structure to inject molecules into the host cell that help reprogram it into a bacterial haven where the pathogen can replicate (Galán, 2021; Dos Santos et al., 2020). Enclosed in a specialized compartment called the *Salmonella*-containing vacuole (SCV), the bacteria can replicate while evading many immune defenses (Li et al., 2023). If *Salmonella* escape the vacuole and invade the cytosol they ultimately expose themselves to the full fury of the host’s immune arsenal.

At the heart of the immune response lies the inflammasome, a molecular sensor that detects when the cell’s inner sanctum is breached. This, in turn, sets off an explosive immune chain reaction. In macrophages, inflammasomes activate inflammatory enzymes, specifically caspase-1 and caspase-4. Caspase-1 triggers the release of alarm signals, such as interleukin-1β (IL-1β), which alert neighboring cells. In tandem, it activates gasdermin D (GSDMD), a pore-forming protein central to initiating a type of programmed cell death known as pyroptosis (Egan et al., 2023a). How exactly inflammasomes restrict bacterial replication has so far been unclear. Now, in eLife, Sunny Shin and colleagues at the University of Pennsylvania – including Marisa Egan as first author – report how two caspases play distinct roles in the battle against *Salmonella enterica* serovar Typhimurium (Egan et al., 2023b).

Using chemical inhibitors and genetic knockouts in human macrophages, Egan et al. showed that, early in the battle, caspase-1 was the frontline warrior (Figure 1). Blocking this enzyme caused IL-1β secretion and pyroptosis to be significantly reduced, allowing *Salmonella* to multiply rapidly within macrophages. As the infection advanced, caspase-4 became more important. In its absence, cells had a higher number of bacteria 24 hours following infection. This finding hints at a strategic, time-sensitive role for caspase-4 – perhaps targeting *Salmonella* that persist within vacuoles or those that have shed their protective layer and moved into the cytosol.

**Figure 1.**
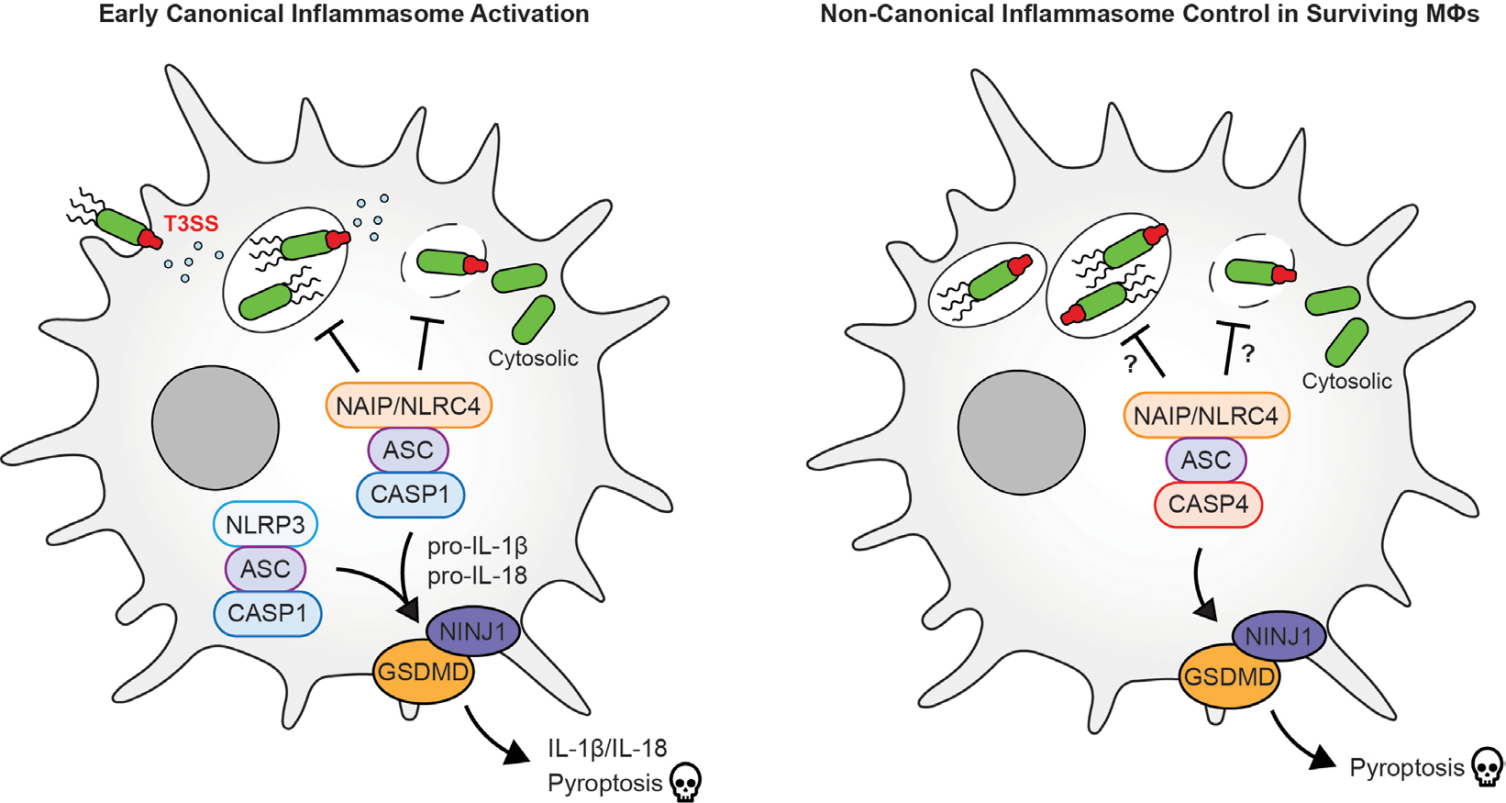
Early and late immune responses of macrophages to *Salmonella* bacteria. (**Left) Early inflammasome activation:** During the early stages of infection, *Salmonella* bacteria use a type III secretion system (T3SSs; green/red structure) to inject virulence factors into the host cell to help it replicate. This, in turn, activates immune responses, such as a molecular sensor called the NAIP/NLRC4 inflammasome, which recognizes components of the T3SS. This leads to the recruitment of an adaptor protein apoptosis-associated speck-like protein containing CARD, called ASC, which is crucial for the formation of various inflammasomes and the activation of the caspase-1 (CASP1) enzyme, which transforms inactive cytokine precursors, notably interleukin-1β (IL-1β) and IL-18, into their active forms, which alert neighboring cells. CASP1 further cleaves gasdermin D (GSDMD), a pore-forming protein, allowing cytokines to be released and leading to pyroptosis, a lytic form of inflammatory cell death. NINJ1, a membrane protein, facilitates the final rupture of the cell membrane. Additional activation of NLRP3, another inflammasome sensor, further amplifies this inflammatory response. (**Right) Late inflammasome control:** During a later stage of infection, caspase-4 (CASP4) takes the lead, and similar to CASP1, cleaves GSDMD and triggers pyroptosis. More research is needed to find out if there is a specific control of *Salmonella* replication in the vacuole and cytosol at this point during infection.

Image credit: Denise Monack, with special thanks to Jeff Hsiao for providing help in making the figure (CC BY 4.0).

The researchers then looked at GSDMD and another protein involved in the final stages of pyroptosis, ninjurin-1 (NINJ1; Fu et al., 2024). When GSDMD was rendered inactive, cytokines were also inactive and cell death was stalled, enabling *Salmonella* to thrive. Similarly, experiments deactivating NINJ1 revealed that even a partial loss of this defense system leads to more bacteria, underscoring its vital role in curbing infection.

One of the most impactful discoveries was that inflammasomes are especially potent at limiting *Salmonella* replication in the cytosol. In a series of cutting-edge experiments – ranging from chemical assays to single-cell microscopy and electron microscopy – Egan et al. uncovered that when inflammasome components like caspase-1 were disabled, the number of *Salmonella* bacteria within the cytosol exploded, and the replication of *Salmonella* within vacuoles also increased significantly. In contrast, intact inflammasome signaling kept the bacterial numbers in check, ensuring their cytosolic presence remained minimal. Their findings underscore the pivotal role of inflammatory caspases and pyroptosis in steering inflammasome responses that confine *Salmonella* replication to specific subcellular niches within human macrophages, a crucial battleground where the pathogen must be contained.

These insights redefine our understanding of host-pathogen warfare and catapult us into the heart of a microscopic war, where inflammasomes orchestrate a ruthless defense against *Salmonella*. The differential roles of caspase-1 and caspase-4 and the concerted actions of GSDMD and NINJ1 provide a detailed map of how our cells fight back against intracellular invaders. This battle provides a fascinating glimpse into the complexities of the human immune response.

By mimicking or enhancing these natural defense mechanisms, we may one day devise treatments that better protect against *Salmonella* and similar pathogens. Human macrophages prove to be a force to be reckoned with, and the striking contrast between human and murine macrophages – where the latter seem less capable of curbing *Salmonella* replication – underscores the importance of studying human cells directly (Thurston et al., 2016). The detailed dissection of these mechanisms offers a thrilling promise for targeted medicine, where boosting the right component of the immune response could tip the scales in favor of the host.
